# Autosomal dominant mutation in *COL7A1* gene causing epidermolysis bullosa dystrophica

**DOI:** 10.1186/1755-8166-7-S1-P58

**Published:** 2014-01-21

**Authors:** Jayesh Sheth, Mehul Mistri, Harsh Patel, Chitra Ankleshwaria, Aradhana Parikh

**Affiliations:** 1 FRIGE’s Institute of Human Genetics, FRIGE House, Ahmedabad, Gujarat, India

**Keywords:** Epidermolysis Bullosa Dystrophica, *COL7A1* gene, c.6712 G>A (p.G2043R) mutation

## Background

Epidermolysis bullosa dystrophica is a rare genetic disease involving skin fragility, resulting in blistering of the skin either spontaneously or following minor skin contact, or trauma. Mutations in COL7A1 gene are associated with all forms of dystrophic epidermolysis bullosa. The gene which is present on the short arm of chromosome 3, codes for a protein called Collagen alpha-1(VII) chain which functions as an anchoring fibril between the external epithelia and the underlying stroma. The aim was to examine a de novo Mutation in Gene causing Epidermolysis bullosa dystrophica in a thirty year old female patient.

## Materials and methods

The case of a thirty-year-old female patient was analyzed. The patient had vesiculobullous lesions, peeling of skin on pressure, milia formation and dorsa of both hands. Lichenified lesions with few fresh bullae were present over the knees and elbows. The histopathological examination was carried out for skin biopsy samples. For the molecular analysis, a sequence study of exons 73 to 75 of the COL7A1 gene was conducted.

## Results

The histopathological examination of the skin biopsy revealed separation of epidermis at dermoepidermal junction along with acanthosis, hyperkeratosis and focal parakeratosis of the overlying epidermal layer. Dermis revealed moderate inflammatory cell infiltration. The immunofluorescence study was negative for IgG, IgM, IgA and C3. These findings were consistent with clinical diagnosis of EB Dystrophica.

Molecular Analysis showed that the patient was heterozygous for c.6712 G>A (p.G2043R) mutation in exon 73 of the COL7A1 gene. Interestingly, the patient had no family history of the disease indicating that the mutation was de novo.

## Conclusion

It is one of the rare examples of a patient who was heterozygous for the mutation with dominant type of EB dystrophy.

